# Loop-Mediated Isothermal Amplification Assay for Identifying Neisseria gonorrhoeae Nonmosaic *penA-*Targeting Strains Potentially Eradicable by Cefixime

**DOI:** 10.1128/spectrum.02335-22

**Published:** 2022-08-24

**Authors:** Ken Shimuta, Hideyuki Takahashi, Yukihiro Akeda, Shu-ichi Nakayama, Makoto Ohnishi

**Affiliations:** a Department of Bacteriology I, National Institute of Infectious Diseases, Tokyo, Japan; b Antimicrobial Resistance Research Centre, National Institute of Infectious Diseases, Tokyo, Japan; c National Institute of Infectious Diseases, Tokyo, Japan; Johns Hopkins Hospital

**Keywords:** cefixime, loop-mediated isothermal amplification, *Neisseria gonorrhoeae*, sexually transmitted infections, *penA*

## Abstract

Treatment regimens for gonorrhea have limited efficacy worldwide due to the rapid spread of antimicrobial resistance. Cefixime (CFM) is currently not recommended as a first-line treatment for gonorrhea due to the increasing number of resistant strains worldwide. Nonetheless, Neisseria gonorrhoeae strains can be eradicated by CFM at a 400 mg/day dose, provided that the strains are CFM responsive (MIC ≤ 0.064 mg/L). To develop a nonculture test for predicting the CFM responsiveness of N. gonorrhoeae strains, we developed an assay to detect N. gonorrhoeae nonmosaic *penA* using loop-mediated isothermal amplification (LAMP). To avoid false-positive reactions with commensal *Neisseria* spp. *penA*, we amplified specific regions of the N. gonorrhoeae *penA* (NG-*penA*-LAMP1) and also the nonmosaic N. gonorrhoeae *penA* (NG-*penA*-LAMP3). This assay was validated using isolated N. gonorrhoeae (*n* = 204) and *Neisseria* spp. (*n* = 95) strains. Clinical specimens (*n* = 95) with confirmed positivity in both culture and real-time PCR were evaluated to validate the system. The combination of the previously described NG-*penA*-LAMP1 and our new NG-*penA*-LAMP3 assays had high sensitivity (100%) and specificity (100%) for identifying N. gonorrhoeae carrying the nonmosaic type. To determine whether CFM could be applicable for gonorrhea treatment without culture testing, we developed a LAMP assay that targets *penA* allele-specific nonmosaic types for use as one of the tools for point-of-care testing of antimicrobial resistance.

**IMPORTANCE** Neisseria gonorrhoeae is among the hot topics of “resistance guided therapy,” one of the top 5 urgent antimicrobial threats according to the Centers for Disease Control and Prevention (CDC). There is a need either to develop new agents or to make effective use of existing agents, with the current limited number of therapeutic agents available. Knowing the drug susceptibility information of the target microorganism prior to treating patients is very useful in selecting an effective antibiotic, especially in gonococcal infections where drug resistance is prominent, and is also important in preventing treatment failure. In this study, we developed a new method for obtaining drug susceptibility profiles of Neisseria gonorrhoeae using the loop-mediated isothermal amplification (LAMP) method. The LAMP assay does not require expensive devices. Therefore, this method is expected to be a tool for point-of-care testing of antimicrobial resistance for individualized treatment in the future.

## INTRODUCTION

Gonorrhea, caused by Neisseria gonorrhoeae, is one of the most common sexually transmitted diseases globally (1–3). The recent increase in multidrug-resistant N. gonorrhoeae has made the choice of drugs for its treatment more difficult. Ceftriaxone (CRO) is the first-line treatment in many countries (3), but it may cease to be an option if the emergence and spread of CRO-resistant strains continues. Employing other drugs in combination with CRO can extend the period during which it is effective, and thus, individualized treatment has been proposed as a potential approach to overcome the problem of resistance (4, 5). This would require monitoring the emergence of resistance, but there is currently no easy assay to estimate gonorrheal susceptibility to CRO. Due to these hurdles, ciprofloxacin (CIP) has recently been recommended in the British Association of Sexual Health and HIV (BASHH) guidelines as a first-line treatment instead of CRO when susceptibility of the strain to CIP is confirmed prior to treating patients (6).

There is no commercially available kit for predicting CRO susceptibility despite numerous studies, while the ResistancePlus GC kit is commercially available for identifying CIP-resistant strains that harbor the S91 mutation and is the only assay currently available commercially for predicting the antibiotic sensitivity of N. gonorrhoeae (7). The low antibiotic sensitivity of some N. gonorrhoeae strains that has been reported in many regions has created an unmet need for treatments other than CIP and CRO (1–3).

Cefixime (CFM), a previously recommended antimicrobial agent for treating gonorrhea, may be considered a therapeutic option against N. gonorrhoeae due to its established effectiveness (8–11). Moreover, orally administered CFM is tolerated by patients and accepted by clinicians. However, CFM is not clinically applicable at present because of decreased susceptibility or resistance to CFM in 24 (47%) of 51 reporting countries in the WHO global antimicrobial resistance surveillance for N. gonorrhoeae 2017 to 2018 and decreased susceptibility or resistance to CFM continuing to emerge in many countries (3). Various third-generation cephalosporin-resistant (or reduced susceptibility) strains (including CFM-resistant strains) carry a mosaic *penA* gene, which encodes penicillin-binding protein 2 (12–14). Mosaic *penA* is formed through natural transformation by the acquisition of genomic DNA from *Neisseria* spp. (15). Furthermore, some cases of CFM treatment failure are attributable to the prevalence of strains carrying the mosaic *penA* gene (16–18). To prevent treatment failure, it is necessary to characterize the drug susceptibility of N. gonorrhoeae strains in individual patients before treatment initiation; however, this approach requires considerable time when using standard culture methods, which would need to be significantly improved for a point-of-care test (POCT). Nonetheless, it would be difficult to accurately characterize third-generation cephalosporin-resistant (or reduced susceptibility) strains based on only a single-gene single nucleotide polymorphism (SNP), including *penA*, because many factors other than solely *penA* mutations are likely to mediate antibiotic resistance (19). Although nucleic acid amplification testing (NAAT) assays that detect several different genetic mutations, including *penA*, have been proposed (20–22), detection systems for third-generation cephalosporin resistance (or reduced susceptibility) are not commercially available.

Conversely, N. gonorrhoeae strains that exhibit a CFM MIC of ≤0.064 mg/L can still be eradicated by high-dose CFM treatment (400 mg/day) (10, 11). Identifying infections caused by strains with a CFM MIC of ≤0.064 mg/L would allow individualized patient stratification for administering effective treatment with CFM.

Among strains isolated in the United Kingdom during 2013 through 2016 (1,266 strains available for analysis from 1,277 isolates), all strains carrying nonmosaic *penA* had a CFM MIC of ≤0.064 mg/L (*n* = 1,175), whereas all 36 N. gonorrhoeae strains with a CFM MIC of >0.064 mg/L carried mosaic *penA* (23). A similar result was observed in 2003 to 2017 isolates from Portugal (24). Furthermore, among the 204 strains of N. gonorrhoeae isolated in Japan (25), 114 had a CFM MIC of >0.064 mg/L and carried mosaic *penA.* All strains carrying nonmosaic *penA* (*n* = 65) exhibited a CFM MIC of ≤0.064 mg/L. Therefore, exempting patients infected by N. gonorrhoeae strains that carry mosaic *penA* from receiving CFM treatment could facilitate effective CFM utilization. In other words, detection of nonmosaic *penA*-carrying strains could increase the usefulness of CFM for treating gonorrhea by avoiding CFM administration in patients predicted to harbor CFM-unresponsive strains.

There is a strong need for a POCT for rapid determination of antimicrobial susceptibility profiles of N. gonorrhoeae because this would facilitate individualized treatment (5). Compared with real-time PCR (RT-PCR) assays, a loop-mediated isothermal amplification (LAMP) assay (26) offers the benefit of a short assay time and a reduced need for specialized laboratory equipment, with potential for use in more remote/resource-constrained regions (27). In previous work, we described a novel LAMP detection system to amplify N. gonorrhoeae *penA* specifically, the NG-*penA*-LAMP1 assay (28).

To this end, in the present study, we developed and evaluated the LAMP assay to identify N. gonorrhoeae nonmosaic *penA*.

## RESULTS

### Correlation between *penA* type and the CFM MIC of N. gonorrhoeae strains.

We investigated the *penA* types of N. gonorrhoeae strains isolated in Japan in 2015 (*n* = 204) (25). We found a total of 17 *penA* types in this sample set (Table 1). Of these, 65 (31.9%) were nonmosaic type, 125 (61.3%) were mosaic type, and 14 (6.9%) were semimosaic type. All strains (*n* = 14) of the semimosaic type belonged to *penA-*150.001. All strains exhibiting a CFM MIC of >0.064 mg/L carried mosaic *penA* (*n* = 114), while all strains carrying nonmosaic or semimosaic *penA* (*n* = 79) exhibited a CFM MIC of ≤0.064 mg/L, although some strains carrying mosaic *penA* (*n* = 11) were also in this group.

**TABLE 1:** LAMP analysis of *penA* genes in the 204 N. gonorrhoeae strains isolated in Japan during 2015

*penA* NG-STAR (no. of strains)	CFM MIC (mg/L)		LAMP result			
			*penA* (NG-*penA*- LAMP1 assay)		Nonmosaic *penA* (NG-*penA*-LAMP3 assay)	
	>0.064	≤0.064	No. positive	No. negative	No. positive	No. negative
1.001_Nonmosaic (16)	0	16	16	0	16	0
2.001_Nonmosaic (6)	0	6	6	0	6	0
2.002_Nonmosaic (14)	0	14	14	0	14	0
5.002_Nonmosaic (18)	0	18	18	0	18	0
9.001_Nonmosaic (3)	0	3	3	0	3	0
10.001_Mosaic (78)	75	3	78	0	0	78
10.008_Mosaic (1)	1	0	1	0	0	1
13.001_Nonmosaic (3)	0	3	3	0	3	0
19.001_Nonmosaic (1)	0	1	1	0	1	0
34.001_Mosaic (1)	1	0	1	0	0	1
60.001_Mosaic (3)	3	0	3	0	0	3
71.001_Mosaic (2)	2	0	2	0	0	2
72.001_Mosaic (19)	19	0	19	0	0	19
101.001_Mosaic (20)	12	8	20	0	0	20
106.001_Nonmosaic (4)	0	4	4	0	4	0
150.001_Semimosaic (14)	0	14	14	0	14	0
152.001_Mosaic (1)	1	0	1	0	0	1
Total strains	114	90	204	0	79	125
*penA type*						
Nonmosaic (65)	0	65	65	0	65	0
Mosaic (125)	114	11	125	0	0	125
Semimosaic (14)	0	14	14	0	14	0
Total strains	114	90	204	0	79	125

### Evaluation of nonmosaic NG-*penA* LAMP detection assays.

We initially investigated the N. gonorrhoeae nonmosaic *penA* primer set developed here using 10-ng genomic DNA samples from two WHO gonococcal reference strains (i.e., WHO G [*penA*-2.001; nonmosaic type] and WHO K [*penA*-10.001; mosaic type]) (29). We verified amplification by the *penA*-LAMP1 primer set of both WHO G (nonmosaic type) and WHO K (mosaic type) sequences. In contrast, a positive reaction was observed only for WHO G (nonmosaic type) when using the nonmosaic *penA* allele (NG-*penA*-LAMP3) but not for WHO K (mosaic type). The detection limit was also determined for the nonmosaic *penA* allele (NG-*penA*-LAMP3) using different amounts of strain WHO G genomic DNA (1 to 1 × 10^6^ genome copies) as the template. A minimum of 1 × 10^4^ genome copies per reaction was detectable using the nonmosaic *penA* allele (NG-*penA*-LAMP3). The detection sensitivity was the same as that of the previously developed *penA*-LAMP1 assay (28).

Next, we investigated the sensitivity and specificity of the NG-*penA*-LAMP3 assay using the genomic DNA (10 ng/reaction) of 204 strains of N. gonorrhoeae and 95 strains of other *Neisseria* spp. that had been previously evaluated using the *penA*-LAMP1 assay (25). DNA amplification using the NG-*penA*-LAMP3 primer set was shown with N. gonorrhoeae strains carrying nonmosaic (*n* = 65) and semimosaic *penA* (*n* = 14) (Table 1). However, N. gonorrhoeae strains carrying mosaic *penA* (*n* = 125) and all *Neisseria* spp., except for Neisseria meningitidis, showed no amplification (Tables 1 and 2).

**TABLE 2:** LAMP analysis of the *Neisseria* species strains

Nongonococcal *Neisseria* species (no.)	LAMP result			
	*penA* (NG-*penA*-LAMP1 assay)		Nonmosaic *penA* (NG-*penA*-LAMP3 assay)	
	No. positive	No. negative	No. positive	No. negative
*N. oralis* (2)	0	2	0	2
*N. mucosa* (28)	0	28	0	28
N. polysaccharea *polyphyletic* (3)	0	3	0	3
*N. subflava* (53)	0	53	0	53
*N. cinerea* (6)	0	6	0	6
N. lactamica (2)	0	2	0	2
N. meningitidis (1)	0	1	1	0

Therefore, positive results from the two assays NG-*penA*-LAMP1 and NG-*penA*-LAMP3 identify N. gonorrhoeae nonmosaic *penA* because NG-*penA*-LAMP1 is negative for N. meningitidis. Consequently, the combination of both the assays had high sensitivity (100%) and specificity (100%) for identifying N. gonorrhoeae nonmosaic-type *penA.*

### Evaluation of LAMP reactions using clinical specimens.

Next, we investigated the efficacy of NG-*penA*-LAMP3 detection in 101 clinical specimens. In our previous work, a primer set for NG-*penA*-LAMP1 was shown to be effective for clinical specimens (28). The 95 clinical specimens were positive, and 6 clinical specimens were negative for N. gonorrhoeae by real-time PCR. All 95 N. gonorrhoeae samples were positive in the NG-*penA*-LAMP1 assay, and all 6 PCR-negative clinical specimens were negative. While 39 of the 95 N. gonorrhoeae specimens were positive, 56 (and the 6 negative controls) were negative in the NG-*penA*-LAMP3 assay. PCR followed by Sanger sequencing confirmed that the 39 NG-*penA*-LAMP3-positive specimens contained nonmosaic *penA*, while mosaic *penA* was present in the 56 NG-*penA*-LAMP3-negative specimens (Table 3). There were no discrepancies whatsoever between these results and those of the NG-*penA*-LAMP3 reactions (Table 3). These results indicate that the NG-*penA*-LAMP-CFM assay accurately distinguishes nonmosaic from mosaic types in clinical specimens. The *penA* data from the 95 N. gonorrhoeae strains are given in the supplemental material (see Table S2 in the supplemental material).

**TABLE 3:** Evaluation of the LAMP assay in real-time PCR-positive specimens

LAMP result	No. of strains			
	*penA* (NG-*penA*-LAMP1 assay)		Nonmosaic *penA* (*penA*-LAMP3 assay)	
	No. positive	No. negative	No. positive	No. negative
*penA* type				
Nonmosaic *penA*	39	0	39	0
Mosaic *penA*	56	0	0	56

### Verification of the relationship between CFM MIC and the *penA* type of N. gonorrhoeae strains in clinical specimens.

**FIG 1:**
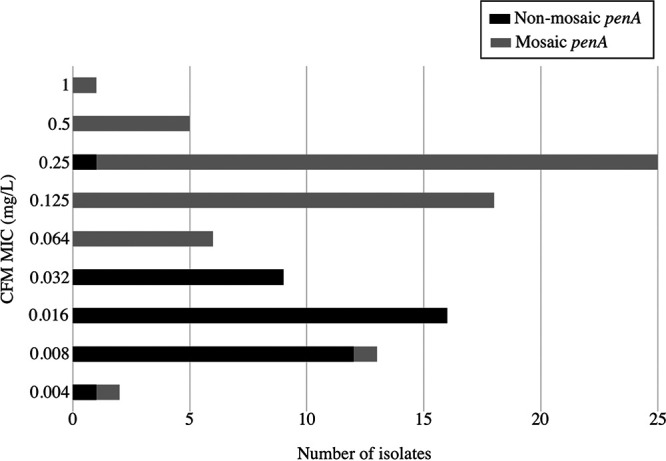
Relationship between the CFM MIC and *penA* type of N. gonorrhoeae strains from clinical specimens. The vertical and the horizontal axes show the CFM MIC value and the number of isolates, respectively. The black and gray bars represent nonmosaic *penA* and mosaic *penA*, respectively.

To confirm that NG-*penA*-LAMP3 results can contribute to decision-making regarding the prescription of CFM, we examined the correlation between CFM MICs and the *penA* types of strains obtained from real-time PCR-positive clinical specimens (Fig. 1). Of the 39 N. gonorrhoeae strains carrying nonmosaic *penA*, 38 (97.4%) exhibited a CFM MIC of ≤0.064 mg/L (Fig. 1; see also Table S2). All of these clinical specimens were positive in the NG-*penA*-LAMP3 assay (Table 3). Reciprocally, of the 46 isolated N. gonorrhoeae strains with a CFM MIC of ≤0.064 mg/L, only 8 (17.4%) carried mosaic NG-*penA* (Fig. 1; see also Table S2). Furthermore, of the 49 N. gonorrhoeae strains with a CFM MIC of >0.064 mg/L, almost all (98.0%) carried mosaic *penA*, while only one harbored the nonmosaic *penA* type (Fig. 1; see also Table S2). From these results, we propose that it is possible to detect N. gonorrhoeae strains carrying nonmosaic *penA* for the purpose of identifying those with a CFM MIC of ≤0.064 mg/L. Therefore, the NG-*penA*-LAMP3 assay may be considered valid for detecting N. gonorrhoeae strains exhibiting a CFM MIC of ≤0.064 mg/L in this study. Thus, this assay will contribute to decision-making as to whether the patient’s N. gonorrhoeae strain will respond to treatment with CFM.

## DISCUSSION

The WHO recommendations currently being followed in multiple regional treatment regimens for gonococcal infections should not be followed when the rate of resistance to first-line drugs in a region exceeds 5% (30). As a consequence, CRO is now being employed as a first-line option in many regions (3). However, in the past decade, CRO-resistant N. gonorrhoeae strains have been reported (2). Agents other than CRO are needed for gonorrhea treatment, which would also prevent the undesired emergence of resistant strains and prolong the useful life of CRO treatment (4). Hence, antimicrobial stewardship is crucial for the control of gonorrhea (2), and there is a growing need for individualized treatment to address these issues (4, 5). Rapid detection systems will provide a valuable point-of-care contribution to clinical decisions on antibiotic use. The establishment of an antimicrobial resistance POCT could provide first-line treatment only to those patients who truly need that specific antibiotic (4, 5). Therefore, here, we pursued a new method to obtain a means of selecting antimicrobial agents that does not depend on conventional empirical treatment.

In this study, we developed a LAMP assay for the detection of N. gonorrhoeae strains with a CFM MIC of ≤0.064 mg/L that correlates with CFM-responsive strains (at 400 mg/day) (10, 11). N. gonorrhoeae strains that exhibit a CFM MIC of ≤0.064 mg/L tend to carry the nonmosaic *penA* type mutation (23, 24). Among N. gonorrhoeae strains with a CFM MIC of ≤0.064 mg/L that were isolated in Japan in 2015 (*n* = 90) were 65 strains (72.2%) that carried nonmosaic *penA*, 14 (15.6%) that carried semimosaic-type *penA*, and 11 (12.2%) that carried the mosaic-type *penA* (Table 1). In contrast, all 114 N. gonorrhoeae strains that exhibited a CFM MIC of >0.064 mg/L carried the mosaic *penA* (25). Therefore, we hypothesized that the detection of nonmosaic *penA* could help identify strains that are susceptible to CFM. Real-time PCR tests to detect nonmosaic or mosaic *penA* type have been developed but require expensive equipment and are unsuitable as POCT assays (20, 21, 31–33). Therefore, POCT technology may facilitate individualized treatment of gonococcal infections (5). In this study, we developed a LAMP assay that can be applied as a POCT to determine the CFM responsiveness of a strain of N. gonorrhoeae.

The NG-*penA*-LAMP1 assay generated the same results as commercial real-time PCR for detecting N. gonorrhoeae strains. However, the NG-*penA*-LAMP3 assay evaluated in the present study showed 100% sensitivity and specificity for discriminating nonmosaic- from mosaic-type *penA*. Furthermore, this novel method could be utilized for the identification of N. gonorrhoeae strains exhibiting a CFM MIC of ≤0.064 mg/L in clinical specimens. Based on the novel assay-based pretesting in this study population, CRO use could be reduced to 40% by replacing first-line CRO with CFM treatment.

Some N. gonorrhoeae strains with mosaic-type *penA* also exhibited a CFM MIC of ≤0.064 mg/L (Table 1; see also Table S2 in the supplemental material) (23–25, 34). Patients infected with these strains carrying mosaic-type *penA* would probably be prescribed CRO as the first-line treatment, in concordance with basic treatment guidelines in many regions (3). However, in regions where *penA* 60.001-associated CRO-resistant strains continue to be isolated (35), if an assay system that can identify the indicator PBP2-311 mutant can be performed simultaneously (28) and those strains are identified, this would also help prevent the spread of *penA* 60.001-associated strains. Additionally, reciprocally, only one of the N. gonorrhoeae strains with a CFM MIC of 0.25 mg/L carried nonmosaic *penA*; it would be difficult to predict whether this strain would be eradicated by cefixime. However, the CLSI breakpoint for N. gonorrhoeae for CFM is S ≤ 0.25 mg/L; there is not a resistance breakpoint (36). The EUCAST breakpoint for N. gonorrhoeae for CFM is S ≤ 0.125 mg/L (37). Thus, to obtain more clinical utility for the N. gonorrhoeae strains exhibiting MIC values of ≤0.064 mg/L in this assay, further validation is required.

We confirmed the 375th to 377th amino acid sequence of PenA from 29 published semimosaic-type PenA sequences (NG-STAR; https://ngstar.canada.ca/) (38) and found 22 semimosaic-type *penA* genes coding GAE as well as the nonmosaic PenA. Therefore, it is expected that these semimosaic types such as *penA* 150.001 may also be detectable by the NG-*penA*-LAMP3 assay. However, 7 other semimosaic type *penA* coded for TPK (*n* = 4), SSK (*n* = 2), and APE (*n* = 1) in the 375th to 377th amino acid sequence of PenA. These semimosaic-type PenA and the mosaic PenA would be expected to not test positive in the NG-*penA*-LAMP3 assay because in this developed assay system, the backward inner primer (BIP) is designed to specifically recognize the nonmosaic-type *penA* genes coding GAE. However, the frequency of strains with semimosaic characteristics is low, and limited information is available on their frequency in certain regions (23–25, 34, 39). Further evaluation will be required to validate the LAMP assay using clinical specimens.

Furthermore, cefixime resistance does not appear to be simple. Other nonmosaic *penA* (PBP2) amino acid substitutions, including at positions 501, 542, and 551, have also been implicated (40–42). In particular, holding *mtrR* and *porB* mutations, strains exhibiting the mutation at position 501 could contribute to cephalosporin resistance (41). In the 2015 panel evaluated in this study, among *penA* 13.001 (nonmosaic *penA*) and *penA* 101.001 (mosaic *penA*) were 501 mutation types. However, all *penA* 13.001 samples had CFM MIC values of ≤0.064 mg/L despite retaining the 501 mutation (Table 1). Conversely, in *penA* 101.001, 8 strains showed a CFM MIC of ≤0.064 mg/L, and 12 strains showed CFM MIC values of >0.064 mg/L. Incorporating an assay system that can simultaneously identify the PBP2-501 mutant may enhance the detection of strains with MIC values of ≤0.064 mg/L in certain regions where PBP2-501 mutant strains with CFM MIC values of >0.064 mg/L are isolated (34, 43).

The novel assay evaluated in this study can most efficiently detect CFM-treatable N. gonorrhoeae strains in populations that mainly have isolated NG strains carrying nonmosaic *penA* with a CFM MIC of ≤0.064 mg/L. These strains were frequently isolated in the United Kingdom and Portugal (23, 24). Moreover, all strains isolated in South Africa in 2018 to 2019 (*n* = 27) exhibited a CFM MIC of ≤0.064 mg/L and carried nonmosaic *penA*, but there is limited region-specific population-level research from South Africa (44). In these regions, considering the characteristics of the isolated strains, the assay described in the present study would likely have been effective in efficiently detecting the strains carrying nonmosaic *penA* that had a CFM MIC of ≤0.064. This could help to enhance the effective use of CFM as a treatment for gonorrhea in these regions. In this context, our approach is expected to be effective in real-world clinical settings worldwide. In certain regions, multiple strains carrying mosaic *penA* have been isolated (45), and other studies have also reported some strains carrying nonmosaic *penA* that had a CFM MIC of >0.064 mg/L (34, 39, 43, 46). There are regional differences in drug susceptibility and *penA* type of the isolated strains of N. gonorrhoeae (3, 39), and several factors other than solely the *penA* type are predicted to be involved in elevating the CFM MIC (19). Therefore, it will be necessary to confirm the characteristics of the N. gonorrhoeae strains in the specific populations tested to allow accurate judgment of the utility of our assay prior to adoption. Constructing a regional map of the characteristics of these strains by monitoring both whole-genome sequencing (WGS) data analysis of isolated strains and surveillance of their antibiotic resistance will help determine whether the novel assay can make a real contribution.

In this newly developed assay, the detection sensitivity was 1 × 10^4^ genome copies per reaction, which is lower than that of real-time PCR methods (47). However, practically, gonococcal bacterial load from urethral specimens for gonorrhea with asymptomatic and symptomatic gonococcal urethritis were 2.0 × 10^5^ copies per swab and 3.7 × 10^6^ copies per swab, respectively (48). Furthermore, both the NG-*penA*-LAMP1 and NG-*penA*-LAMP3 assays were validated using swab samples collected from actual patients with suspected urethritis (Table 3). Considering these results, the assay developed in this study would be effective in detecting the gene of N. gonorrhoeae in actual urethritis specimens.

In the work presented here, only N. meningitidis but no other *Neisseria* spp. yielded positive results in our test, presumably because of the high similarity between the N. gonorrhoeae and N. meningitidis *penA* sequences (GenBank accession number AB904141.1) in the primer annealing regions. This problem could be resolved by using a combination of previously developed NG-specific assays, such as NG-*penA*-LAMP1 (28). At present, it appears that the developed assay can be effective to determine the antimicrobial agent for urethritis patients because the developed assay has not been well validated for pharyngeal specimens and CFM can have low effectiveness in patients with pharyngeal gonorrhoeae (49, 50).

NAAT technology is widely used in clinical settings for definitive testing of N. gonorrhoeae (51, 52), but the development of technology that can predict drug susceptibility has lagged behind. ResistancePlus GC (beta) is the only commercially available product that uses NAATs to predict drug susceptibility (7). However, this assay system requires real-time PCR and is therefore unsuitable as a bedside POCT. In contrast, the LAMP assay can be run in resource-limited laboratories and does not require expensive dedicated devices (26, 27, 53). In addition, a LAMP system that enables fast amplification (25 to 35 min) and multiplex detection has been developed recently (54) In the near future, the application of LAMP assays is expected to contribute to individualized treatment for patients with gonorrhea by allowing for POCT.

In conclusion, the novelty of this research is the development of a POCT method for rapid determination of antimicrobial susceptibility profiles of N. gonorrhoeae by LAMP assay, which does not rely on culture for identifying N. gonorrhoeae strains that can be treated with CFM. While further validation using clinical specimens from other global regions is necessary, the introduction of technology based on the assays developed herein could contribute to the enhancement of individualized patient treatment by tailoring antibiotic administration only to patients harboring N. gonorrhoeae strains that are known to be susceptible to that particular antimicrobial agent.

## MATERIALS AND METHODS

### *Neisseria* isolates and antimicrobial susceptibility.

Isolation of the 204 N. gonorrhoeae strains and 95 other *Neisseria* species strains has been previously reported (25, 28, 47). The agar dilution method was used to determine antimicrobial susceptibility according to Clinical and Laboratory Standards Institute protocol (36).

### Clinical samples.

From 2020 to 2021, 101 urethral swabs of male patients with urethritis were collected from the urology clinic as part of our ongoing surveillance program. Confirmation of the N. gonorrhoeae strain contained in the clinical sample was accomplished using real-time PCR (Cobas 4800 System; Roche, Mannheim, Germany), which is employed by clinical laboratories. The N. gonorrhoeae strains were isolated from real-time PCR-positive samples. Antimicrobial drug susceptibility testing was performed to determine the MIC for these isolated strains. Separately, urethritis swab specimens were suspended in 200 μL Tris-EDTA buffer and boiled for 5 min, centrifuged at 9,000 × *g*, and the supernatant stored at −20°C until use (28). Two microliters of supernatant were used as a template for the clinical specimens in this study. The study was approved by the Institutional Review Board of the National Institute of Infectious Diseases (approval number 993).

### DNA extraction.

Genomic DNA was purified using QIAamp DNA minikit (Qiagen, Venlo, The Netherlands) according to the manufacturer’s instructions. Double-stranded DNA concentrations were determined using Qubit double-stranded DNA (dsDNA) HS assay kits (Invitrogen, Carlsbad, CA). The same purified genomic DNA was used for conventional PCR and LAMP assays.

### Development of the nonmosaic N. gonorrhoeae *penA* LAMP detection assay.

Because N. gonorrhoeae *penA* shares sequence similarity with some commensal *Neisseria* spp. *penA* genes (12–15), two independent assays were required to eliminate false-positive reactions with the latter. NG-*penA*-LAMP1 assay, previously described, was developed for amplification of N. gonorrhoeae *penA* specifically, based on the sequence of its 5′-terminal half (55). The 204 N. gonorrhoeae strains (25) and 14 WHO gonococcal reference strains (29) were positive, while the other 95 *Neisseria* species strains were negative in this NG-*penA*-LAMP1 assay (28). This NG-*penA*-LAMP1 was used as an internal control in this study since this amplification assay can be valid specifically for N. gonorrhoeae *penA*.

The 5′-terminal half of the N. gonorrhoeae *penA* sequence is common among gonococci, while the 3′-terminal half of this region contains some sequences with low similarity between nonmosaic and mosaic *penA* (55). In the present study, we attempted to develop a LAMP assay to specifically amplify the nonmosaic *penA* allele (NG-*penA*-LAMP3). Because the NG-*penA*-LAMP3 primer sequence was designed to anneal to regions within the 3′-terminal half of *penA*, it was proposed that the NG-*penA*-LAMP3 assay system would be able to detect the presence of N. gonorrhoeae nonmosaic *penA*.

Thus, positive results of two independent assays indicated the presence of N. gonorrhoeae nonmosaic *penA*.

### LAMP primer design of nonmosaic *penA*.

The 375th to 377th amino acid region has been shown to be different in the nonmosaic- and mosaic-type N. gonorrhoeae PenA amino acid sequence (39, 56), with the former encoding GAE and the latter TPK. Based on this difference, for the primer design, we selected the N. gonorrhoeae *penA* sequence comprising nucleotides 1019 to 1213 of the WHO Reference strain F *penA* (GenBank accession number LT591897.1) from the start codon. The LAMP assay primer sets for this study were designed using Primer Explorer v5 software (https://primerexplorer.jp/v5_manual/021.html; Fujitsu, Tokyo, Japan). The oligonucleotides were designed to be specific for each target (see Fig. S1 in the supplemental material). Specific detection of the nonmosaic form using the NG-*penA*-LAMP3 primer set was dependent on the B1 region (Fig. S1). The 5′ terminus of the BIP primer (B1 region) corresponded to the 375th amino acid residue (glycine). Consequently, a primer set for the NG-*penA*-LAMP3 assay to discriminate nonmosaic-type from mosaic-type *penA* could be generated. The primer sequences were identical to those of *penA-*150.001 (semimosaic) and are shown in Table S1 in the supplemental material.

### LAMP reaction.

The LAMP assay was performed as previously described using a SimpliAmp thermal cycler (Applied Biosystems, Foster City, CA) (28). The LAMP products were visually confirmed using UV fluorescence (53).

### *penA* sequence.

The *penA* alleles in gonococcal strains isolated in 2015 in Japan were extracted from whole-genome sequencing (WGS) data using FA1090 as a reference sequence as previously described (25). These WGS data had been deposited as accession numbers DRX117493 through DRX117696 under the National Center for Biotechnology Information BioProject (number PRJDB6496) (25). The *penA* sequences (WHO G, GenBank accession number LT591898.1; WHO K, GenBank accession number LT591908.1) were extracted from each genome sequence. The *penA* alleles of 2020 and 2021 isolates were confirmed using the conventional Sanger sequencing method as previously described (55). The *penA* sequence of N. gonorrhoeae was assigned using NG sequence typing for antimicrobial resistance (NG-STAR) (38).

### Data availability.

The complete nucleotide sequences of the novel mosaic *penA* gene of 215.001 have been deposited in the DNA Data Bank of Japan under accession number LC671670 (see Table S2 in the supplemental material).
