# Supplementary material for: Loop-Mediated Isothermal Amplification Assay for Identifying Neisseria gonorrhoeae Nonmosaic penA-Targeting Strains Potentially Eradicable by Cefixime
Source: Microbiol Spectr. 2022 Aug 24;10(5):e02335-22. doi: 10.1128/spectrum.02335-22 (PMC9602674; doi:10.1128/spectrum.02335-22)
Supplement: Supplemental file 1 — Figure S1, Table S1, Table S2. Download spectrum.02335-22-s0001.pdf, PDF file, 0.1 MB [file spectrum.02335-22-s0001.pdf]

Supplementary data

Figure S1. Location of *penA*-LAMP3 primer in *Neisseria gonorrhoeae* wild-type *penA* from the WHO Reference strain F (J Antimicrob Chemother 2016; 71: 3096–3108. GenBank: LT591897.1). Each number indicates the position from the start codon of the WHO Reference strain F *penA*. □: Solid boxes show each selected primer. ------: Dashed lines indicate loop primer.

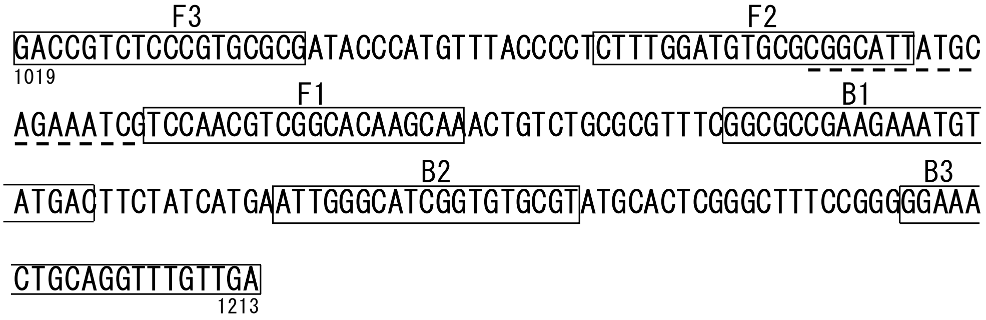

Supplemental Table S1. LAMP primers used in this study

Name	Sequence (5`→ 3`)	Final concentration (μM)	Reference
NG-*penA*-LAMP1			Antimicrob Agents Chemother 2020; 64: e01663-19.
penA-F3	TGACCAGTAACGGACGGATT	0.2	
penA-R3	CTCAACGCCAAAACCGCA	0.2	
penA-FIP	TACCGTCTGCAGATACAGCCCGAATGGCGGTCTTGTTTTCCT	1.2	
penA-BIP	TGAAAGAACAGGGCGACAACCGGCGTGTAGCCGGCAGTGC	1.2	
penA-LF	CGGGCAATCAGACAGGCAA	0.8	
penA-LB	GATTGTGCGGACTCAAGCAT	0.8	
NG-*penA*-LAMP3			Current study
NonMosaic-F3	GACCGTCTCCCGTGCGC	0.27	
NonMosaic-R3	TCAACAAACCTGCAGTTTCC	0.27	
NonMosaic-FIP	TTGCTTGTGCCGACGTTGGACTTTGGATGTGCGCGGCATT	1.2	
NonMosaic-BIP	GGCACCGAAGAAATGTATGACACGCACACCGATGCCCAGT	1.2	
NonMosaic-LF	CGATTTCTGCATAATGCCG	0.8	

Note: The FIP primer was composed of the F2 region at the 3' end and a sequence

complementary to the F1 region at the 5' end. The BIP primer was composed of the B2 region at the 3' end and a sequence complementary to the B1 region at the 5' end. An amplification refractory mutation system (ARMS) strategy (Nucleic Acids Research 1989; 17:2503–2516) was employed to suppress false-positive reactions owing to the presence of a mosaic *penA* type; some nucleotide sequences improved the primer (Table S1). Thus, an intentional mutation was introduced at the fourth or second nucleotide, which is underlined in bold, at the 5′ and/or 3′ terminus of the BIP (B1c+ B2) primers.

Supplemental Table S2. Distribution of *penA* genes in culture-confirmed *N. gonorrhoeae* strains from NAAT-positive clinical specimens

	CFM MIC (mg/L)	
penA NG-STAR (n= no. of strains)	>0.064	≤0.064
1.001 _NonMosaic (n=2)	0	2
2.001_NonMosaic (n=17)	1	16
2.002 _NonMosaic (n=10)	0	10
5.002 _NonMosaic (n=8)	0	8
10.001_Mosaic (n=38)	36	2
10.008 _Mosaic (n=1)	1	0
18.001_NonMosaic (n=1)	0	1
34.001_Mosaic (n=4)	2	2
34.006 _Mosaic (n=2)	1	1

34.007_ Mosaic (n=4)	3	1
72.001_Mosaic (n=2)	1	1
101.001_Mosaic (n=4)	4	0
106.001 _NonMosaic (n=1)	0	1
215.001 _Mosaic (n=1)	0	1
Total strains	49	46
penA type		
(n= no. of strains)		
NonMosaic (n=39)	1	38
Mosaic (n=56)	48	8
Total strains	49	46
